# Dr. Edmund A. Reilly

**Published:** 1913-06

**Authors:** 


Dr. Edmund A. Reilly died at Elmira, N. Y., on April 29, 1913. He was born in Elmira April 6, 1860. Dr. Reilly graduated in 1881 from the Medical Department of Western Reserve University.

